# Analgesic Effect of Extracorporeal Shock Wave Treatment Combined with Fascial Manipulation Theory for Adhesive Capsulitis of the Shoulder: A Retrospective Study

**DOI:** 10.1155/2018/3450940

**Published:** 2018-01-18

**Authors:** Xiangnan Yuan, Fenghua Zhou, Lixin Zhang, Zhiqiang Zhang, Jianjun Li

**Affiliations:** ^1^Rehabilitation Department, Shengjing Hospital of China Medical University, Shenyang, Liaoning 110004, China; ^2^Orthopaedic Department, Shengjing Hospital of China Medical University, Shenyang, Liaoning 110004, China

## Abstract

**Objectives:**

This study aims to explore whether extracorporeal shockwave treatment (ESWT) based on the theory of fascial manipulation (FM) at select treatment points is superior to traditional local ESWT for pain relief in adhesive capsulitis of the shoulder.

**Methods:**

Data from patients with adhesive capsulitis of the shoulder who received weekly ESWT according to fascial manipulation theory (ESWT-FM) or local extracorporeal shockwave treatment (L-ESWT) during a 5-week treatment period were evaluated. Pain-on-movement numeric rating scale (p-NRS) and range of motion (ROM) testing were performed before the treatment period, after the first treatment, and after the fifth treatment.

**Results:**

There were significant reductions in pain scores in the ESWT-FM group (*p* < 0.05) after the first treatment, and after the fifth treatment, both groups had marked, significant improvement (*p* < 0.05), with a significantly greater reduction in pain (p-NRS) in the ESWT-FM group compared to the L-ESWT group (*p* < 0.05). There was no significant difference in terms of ROM in the L-ESWT group, while there was slight improvement of forward flexion in the ESWT-FM group after the fifth treatment.

**Conclusions:**

ESWT-FM provided faster pain relief and slightly more notable improvement of function compared with L-ESWT for the patients with adhesive capsulitis of shoulder.

## 1. Introduction

Shoulder pain is a common musculoskeletal malady, and one of the most prevalent causes of shoulder pain is adhesive capsulitis of the shoulder (AC), which may be associated with minor trauma, environmental stresses, autoimmune processes, or disease like diabetes mellitus and so forth [1, 2]. AC results from inflammation, fibrosis, and contracture of the joint capsule or adjacent bursa, which manifests as a progressive loss of active and passive shoulder movement accompanied by pain [1, 3]. In a retrospective review of 234 patients, 89.5% of AC cases were treated successfully without the need for surgical intervention [4]. Nonsurgical or minimally invasive treatment options for AC include nonsteroidal anti-inflammatory medications, corticosteroid injection at the affected area, hydrodilatation, manipulation under anaesthesia, and physiotherapy [3, 5]. More recently, extracorporeal shockwave therapy (ESWT), as a sort of physical factor, has been proven to be effective for relief of painful shoulder conditions, including AC [6, 7] and supraspinatus tendinopathy [8]. Most of the current literature on ESWT for musculoskeletal disorders has focused on its use in the treatment of bone disorders, including osteonecrosis of femoral head and nonunion of bones [9, 10], and treatment of tendinopathies [10, 11], including lateral elbow epicondylitis [12], plantar fasciopathy [13], calcific tendinitis of shoulder [14], and patellar tendinopathy [15]. Previous ESWT studies have typically focused on application to painful and local treatment points localized in the affected tendon, muscle, or bone [6–8, 10, 11]. And in prior evaluations of ESWT for AC, although the number of studies is very small, the treatment was usually applied only to local tender points also with inconclusive results [6, 7].

During recent years, the critical role of the fascia in the pathogenesis of musculoskeletal pain and dysfunction has gradually been accepted [16], and there is a prevailing view that the myofascial system is a three-dimensional continuum wherein musculoskeletal disorders may be caused by changes in the deep muscle fascia, such as lack of sliding, stretching, and appropriate adaptation. Constant nonphysiological tension in a fascial segment may lead to the formation of adaptive fibroses, which may cause pain both distally and proximally [17]. In keeping with this theory, musculoskeletal dysfunction, including painful shoulder syndrome [16] and TMJ disorders [17], has been treated successfully with the novel treatment strategy of fascial manipulation (FM) at points away from the painful area [18, 19]. Under this theory of FM, determination of the appropriate treatment area for the pain of AC requires consideration of not only the local point of pain but also the related functional muscle and fascia in the surrounding region [18].

The purpose of the present study is to determine, by retrospective review, whether AC-related pain could be more effectively treated by ESWT according to FM theory than by conventional local ESWT alone.

## 2. Materials and Methods

The study included 34 patients who were treated for AC at Shengjing Hospital during the period between January 2015 and July 2017. Patients were included in the study if they were 18 years old or older, exhibited shoulder pain with restriction in ROM of >50% in abduction or flexion and external or internal rotation, experienced symptoms for more than 3 months or had not received treatment, had undergone shoulder radiography, soft tissue sonography, and/or shoulder magnetic resonance imaging a minimum of 14 days prior to selection for ESWT treatment, and did not receive additional pain management procedures, such as intra-articular injection or oral medication, during the therapy. Written informed consent was obtained from every patient before beginning treatments.

Patients were excluded from the study if they were pregnant, if they had had surgical intervention on the affected shoulder, if there was extensive scar around the shoulder, rotator cuff calcification, joint infection, lack of stability, rheumatoid arthritis or full thickness tear of shoulder rotator cuff, cervical radiculopathy or damage to the spinal cord, or history of cortisone injection in the affected area in the previous 6 weeks, or if they had other contraindications to shock wave treatment, including artificial pacemaker, use of anti-blood clotting medications, known bleeding disorder, known malignancy in the area intended for treatment, or epilepsy.

The patients were divided into two groups. All patients underwent 5 sessions of ESWT during each seven-day interval. One group received ESWT according to the fascial manipulation theory (ESWT-FM) and the other had local ESWT (L-ESWT) only. A Swiss DolorClast radial shockwave device (EMS Electro Medical Systems, Nyon, Switzerland) with pressure in the range of 1.5 to 2.5 bars was employed at 0.08 to 0.28 mJ/mm^2^ and 10 to 13 Hz frequency. In the case of the L-ESWT group, the two chosen local tender treatment points were the anterior shoulder joint, with the superior edge of the painful treatment area being just lateral to the coracoid process, and an area that was 1 cm proximal to the tendon attachment to bone. For those in the ESWT-FM group, FM guidelines were followed to choose centers of coordination points based on the physical examination, in addition to the two local tender treatment points [15]. The horizontal plane was often chosen, and the treatment points were at the lower section of the intrarotator muscle insertions at the humerus (IR-Hu); below the elbow crease at pronator teres, for the point with highest sensitivity (IR-CU); at trapezius, immediately above the superior angle of the scapula (ER-SC); and at the posterior aspect of the rotator cuff (ER-HU), laterally to triceps tendon, in the fascia and lateral septum (ER-CU) [16]. Approximately 450 to 500 shocks were applied at every treatment point, according to the patient's tolerance. During the 5-week treatment period, local electrotherapy was administered to all patients as the standard and baseline treatment, consisting of ultra-short-wave therapy, intermediate frequency electrotherapy, or ultrasonic therapy.

Pain scores and basic shoulder functionality were assessed prior to treatment and after the first and fifth treatment sessions, based on the pain-on-movement numeric rating scale (p-NRS), with a range of 0 (no pain) to 10 (severe pain), and range of motion (ROM) testing, which evaluated forward flexion, abduction, and internal and external rotation.

### 2.1. Statistical Analysis

The Statistical Package for the Social Sciences v16.0 (SPSS Inc., Chicago, Illinois) was used for data collection and analysis. Independent samples *t*-test and repeated-measures one-way analysis of variance (ANOVA) were, respectively, used for intergroup and intragroup analyses. Statistical significance was indicated by two-sided *p* values of <0.05.

## 3. Results

There were 16 patients in the ESWT-FM group and 18 patients in the L-ESWT group. The groups did not differ significantly at baseline in terms of affected side, duration of pain, and p-NRS (Table 1).

**Figure 1:**
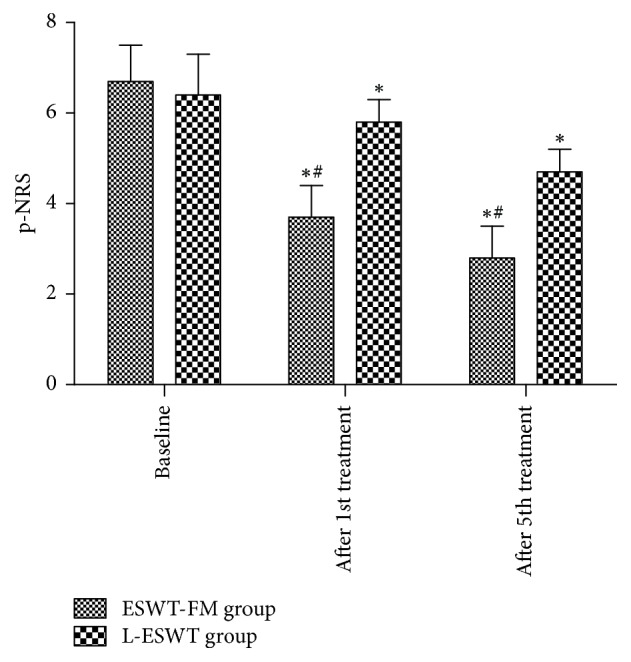
Comparison of p-NRS after ESWT-FM and L-ESWT. ESWT-FM: extracorporeal shockwave therapy combined with fascial manipulation theory; L-ESWT: local extracorporeal shockwave treatment. *∗* indicates comparison of p-NRS after treatment versus baseline, *p* < 0.05; ^#^p-NRS after treatment; comparison between groups, *p* < 0.05.

After the first treatment, p-NRS showed a statistically significant improvement in both groups (*p* < 0.05), and there was significantly more improvement in the ESWT-FM group compared to the L-ESWT group (*p* = 0.0001) (Figure 1). After the fifth treatment, both groups showed remarkable improvement (*p* < 0.05), and again the improvement in p-NRS was significantly greater in the ESWT-FM group compared to the L-ESWT group (*p* = 0.0001) (Figure 1).

We only observed slight significant improvement in forward flexion in the ESWT-FM group after the fifth treatment (*p* = 0.001), and there was a significant difference between groups (*p* = 0.001). There was no significant difference in terms of range of motion in either group other than the improvement in forward flexion in the ESWT-FM group after the first and fifth treatment sessions, and there was no significant difference between groups (Table 2).

## 4. Discussion

We found that both treatment groups experienced pain relief but that the relief was quicker and was more significant after ESWT-FM, both after the first treatment session and after the overall treatment. This finding corroborated the results of earlier studies. When ESWT treatment was compared with oral steroids for treatment of AC, improvements in the total constant shoulder score and in the activities of daily living and ROM parameters of that score were statistically significant in the ESWT group from study commencement to the sixth week, while the pain and power parameters were statistically significant between the second and fourth weeks [6]. While some studies note better results with ESWT [7], others have found only limited efficacy for the treatment of shoulder pain [8, 20]. In the present study, ESWT-FM was associated with a 50% reduction in p-NRS after a single session, suggesting quicker pain relief.

The treatment points chosen in this study were not the same as those in earlier studies. In addition to conventional points around the shoulder (e.g., affected rotator interval and coracohumeral ligament) [1], several centers of coordination points were chosen as well, based on the physical examination and in accordance with the FM guidelines. FM theory construes the myofascial system as a three-dimensional continuum, and musculoskeletal dysfunction occurs when there is lack of sliding, stretching, and appropriate adaptation of the muscular fascia. The shoulder is viewed as part of this interconnected system, and its functionality depends on how it interacts with the other components of the system [17–19]. Issues arising in the shoulder can lead to alterations in the local fascia, which in turn will cause further changes or referred pain in distal or proximal segments (e.g., elbow or wrist joint), while the constant nonphysiological tension in the deep fascia of the affected area can induce the formation of adaptive fibrosis [16]. Therefore, to minimize the likelihood of fibrosis, restore physiological tension in the deep fascia, and facilitate rapid alleviation of pain, distal points over the deep fascia are chosen as treatment points, as they were in this study.

We observed a slight improvement in forward ROM after FM-ESWT. It is known that both pain relief and ROM improvements are possible with therapeutic exercises and mobilization [1, 5], and in the present study we could not distinguish which effects, ESWT-FM or the standard exercise program, contributed most to the ROM improvements. Certainly, pain relief and restored physiological tension in the deep fascia after ESWT-FM may have helped to improve participation in the exercise program. Nonetheless, to determine whether AC recovery is enhanced by a supervised exercise program on its own or combined with ESWT-FM, additional research must be conducted.

Due to its retrospective design, this study could not produce the same high-caliber evidence as a double-blind randomized clinical trial and, moreover, the sample was insufficiently large. Thus, to gain more data regarding the efficiency of ESWT-FM alongside therapeutic exercises and mobilization to achieve long-term pain and ROM improvements in patients with AC, additional prospective randomized blinded controlled clinical trials must be conducted.

## 5. Conclusions

ESWT was applied in this study according to fascial manipulation theory to both local and distal treatment points chosen in keeping with the three-dimensional continuum view of the myofascial system. According to the obtained result, notable pain and slight functionality improvements were achieved through administration of ESWT-FM.

## Figures and Tables

**Table 1:** Baseline clinical characteristics.

	Number (female/male)	Age (year)	Duration of pain (month)	Affected side (left/right)	p-NRS
ESWT-FM group	16 (9/7)	53.6 ± 5.1	4.1 ± 0.6	5/11	6.7 ± 0.8
L-ESWT group	18 (10/8)	52.8 ± 4.9	3.9 ± 0.4	6/12	6.4 ± 0.9

ESWT-FM: extracorporeal shockwave therapy combined with fascial manipulation theory; L-ESWT: local extracorporeal shockwave treatment.

**Table 2:** Comparison of range of motion results after ESWT-FM and L-ESWT.

	Baseline	After 1st treatment	After 5th treatment
*ESWT-FM group*			
Forward flexion	75.1 ± 12.5	81.8 ± 10.3	90.1 ± 9.3^*∗*#^
Lateral abduction	57.9 ± 13.3	62.3 ± 14.5	66.7 ± 15.9
External rotation	10.5 ± 4.1	11.6 ± 4.9	12.4 ± 4.9
Internal rotation	14.8 ± 6.6	16.1 ± 7.4	17.1 ± 8.1
*L-ESWT group*			
Forward flexion	73.7 ± 11.2	75.3 ± 11.9	77.1 ± 11.8
Lateral abduction	56.8 ± 14.7	58.7 ± 14.9	61.5 ± 14.9
External rotation	9.9 ± 4.3	10.5 ± 4.4	11.7 ± 4.6
Internal rotation	15.2 ± 7.1	15.9 ± 7.3	16.9 ± 7.6

ESWT-FM: extracorporeal shockwave therapy combined with fascial manipulation theory; L-ESWT: local extracorporeal shockwave treatment; ^*∗*^range of motion after treatment versus baseline, *p* < 0.05. ^#^Range of motion after treatment; comparison between groups, *p* < 0.05.
